# (*E*)-2-Cyano-*N*′-(1,2,3,4-tetra­hydro­naphthalen-1-yl­idene)acetohydrazide

**DOI:** 10.1107/S160053681202106X

**Published:** 2012-05-16

**Authors:** Mohamed A. Al-Omar, Nagy M. Khalifa, Hazem A. Ghabbour, Tze Shyang Chia, Hoong-Kun Fun

**Affiliations:** a Department of Pharmaceutical Chemistry, College of Pharmacy, King Saud University, Riyadh 11451, Saudi Arabia; b Drug Exploration & Development Chair (DEDC), College of Pharmacy, King Saud University, Riyadh 11451, Saudi Arabia; c Department of Therapeutic Chemistry, Pharmaceutical and Drug Industries Division, National Research Centre, Dokki 12622, Cairo, Egypt; d X-ray Crystallography Unit, School of Physics, Universiti Sains Malaysia, 11800 USM, Penang, Malaysia

## Abstract

In the title compound, C_13_H_13_N_3_O, the tetra­hydro­benzene ring adopts a half-boat (envelope) conformation. The mean plane of the tetra­hydro­naphthalene ring system forms a dihedral angle of 9.56 (6)° with the mean plane of the cyano­acetohydrazide group. In the crystal, inversion dimers linked by pairs of N—H⋯O hydrogen bonds generate *R*_2_^2^(8) loops. The dimers are connected by C—H⋯N hydrogen bonds into a chain propagating along [101]. The crystal packing also features C—H⋯π inter­actions.

## Related literature

For background to tetra­lin, see: Dutta *et al.* (2002 ▶); Taddei *et al.* (2002 ▶); Zaghary *et al.* (2005 ▶); Bahgat & Khalifa (2006 ▶); El Nezhawy *et al.* (2009 ▶); Khalifa *et al.* (2008 ▶). For ring puckering parameters, see: Cremer & Pople (1975 ▶).
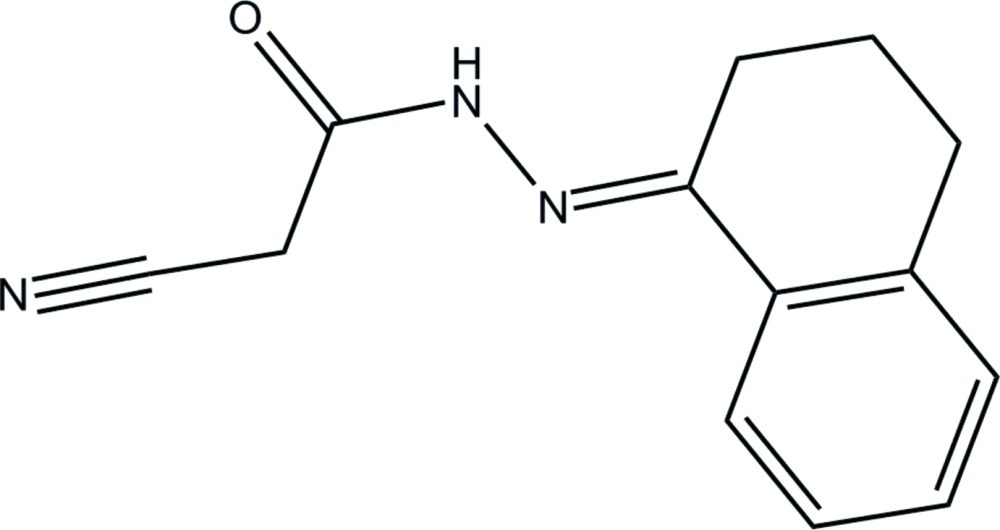


## Experimental

### 

#### Crystal data

C_13_H_13_N_3_O
*M* *_r_* = 227.26
Triclinic, 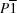

*a* = 7.6414 (1) Å
*b* = 7.6748 (1) Å
*c* = 10.5644 (2) Å
α = 109.589 (1)°
β = 91.405 (1)°
γ = 93.260 (1)°
*V* = 582.13 (2) Å^3^
*Z* = 2
Cu *K*α radiation
μ = 0.69 mm^−1^
*T* = 296 K
0.59 × 0.51 × 0.40 mm

#### Data collection

Bruker SMART APEXII CCD diffractometer
Absorption correction: multi-scan (*SADABS*; Bruker, 2009 ▶) *T*_min_ = 0.687, *T*_max_ = 0.771
5749 measured reflections
1898 independent reflections
1762 reflections with *I* > 2σ(*I*)
*R*_int_ = 0.017

#### Refinement

*R*[*F* ^2^ > 2σ(*F* ^2^)] = 0.043
*wR*(*F* ^2^) = 0.120
*S* = 1.05
1898 reflections
159 parameters
H atoms treated by a mixture of independent and constrained refinement
Δρ_max_ = 0.18 e Å^−3^
Δρ_min_ = −0.17 e Å^−3^

### 

Data collection: *APEX2* (Bruker, 2009 ▶); cell refinement: *SAINT* (Bruker, 2009 ▶); data reduction: *SAINT*; program(s) used to solve structure: *SHELXTL* (Sheldrick, 2008 ▶); program(s) used to refine structure: *SHELXTL*; molecular graphics: *SHELXTL*; software used to prepare material for publication: *SHELXTL* and *PLATON* (Spek, 2009 ▶).

## Supplementary Material

Crystal structure: contains datablock(s) global, I. DOI: 10.1107/S160053681202106X/hb6781sup1.cif

Structure factors: contains datablock(s) I. DOI: 10.1107/S160053681202106X/hb6781Isup2.hkl

Supplementary material file. DOI: 10.1107/S160053681202106X/hb6781Isup3.cml

Additional supplementary materials:  crystallographic information; 3D view; checkCIF report

## Figures and Tables

**Table 1:** Hydrogen-bond geometry (Å, °) *Cg*1 is the centroid of the C5–C10 ring.

*D*—H⋯*A*	*D*—H	H⋯*A*	*D*⋯*A*	*D*—H⋯*A*
N1—H1*N*1⋯O1^i^	0.91 (2)	1.96 (2)	2.8640 (17)	174.7 (19)
C10—H10*A*⋯N3^ii^	0.93	2.58	3.491 (3)	167
C2—H2*A*⋯*Cg*1^iii^	0.97	2.80	3.6775 (17)	152

Symmetry codes: (i) 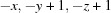; (ii) 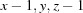; (iii) 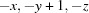.

**Fig. 1.**
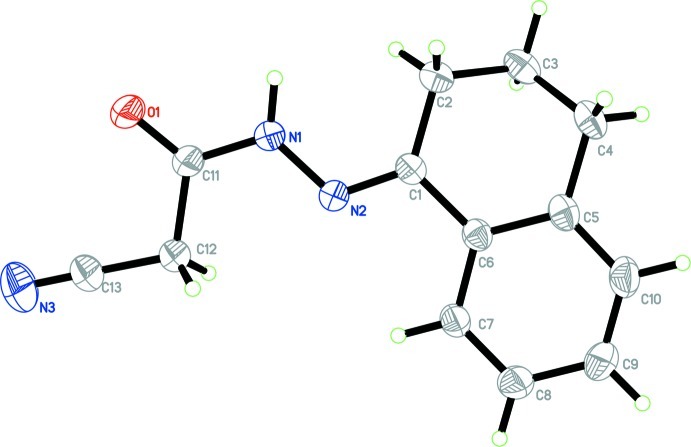
The molecular structure of the title compound with 30% probability displacement ellipsoids.

**Fig. 2.**
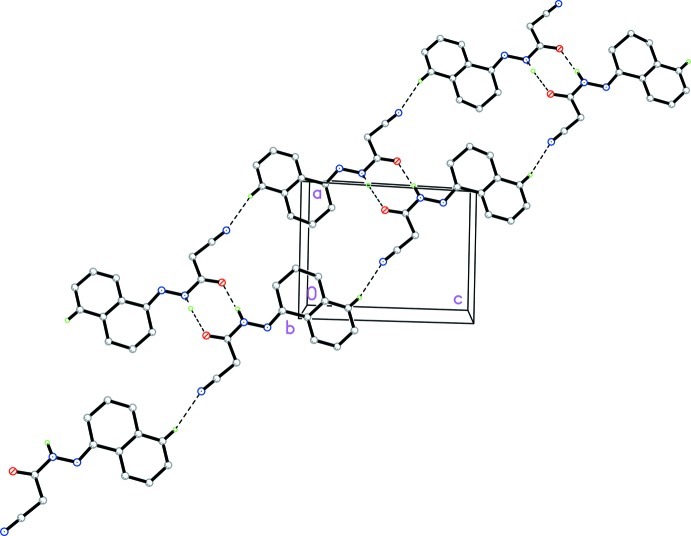
The crystal packing of the title compound. The dashed lines represent the hydrogen bonds. For clarity sake, hydrogen atoms not involved in hydrogen bonding have been omitted.

## supplementary crystallographic information

### Comment

Tetralins (tetrahydronaphthalene derivatives) are of increasing interest since many of these compounds play a vital role in the biological activities because of their biological potentialities, for example, as potent agonists for D2-type receptors (Dutta *et al.*, 2002), a treatment of Alzheimer's disease (Taddei *et al.*, 2002) and as anti-cancer agents (Zaghary *et al.*, 2005). Also, we found that certain substituted tetralin and heterocyclic derivatives show inhibition for cercarial serine protease (Bahgat & Khalifa, 2006), antioxidant (El Nezhawy *et al.*, 2009) and antiinflammatory (Khalifa *et al.*, 2008) activities. Tetralin derivative containing cyanoaceto-hydrazide was prepared as the title compound and its crystal structure is now reported.

The asymmetric unit of the title compound is shown in Fig. 1. The tetrahydrobenzene ring (C1–C6) adopts a half-boat conformation with puckering parameters (Cremer & Pople, 1975), *Q* = 0.4695 (16) Å, θ = 122.4 (2)° and φ = 308.6 (2)°. The flap atom C3 deviates from the mean plane of C1/C2/C4–C6 by -0.6395 (16) Å. In the molecule, the mean plane of tetrahydronaphthalene ring system (C1–C10) forms a dihedral angle of 9.56 (6)° with the mean plane of cyanoacetohydrazide group [O1/N1–N3/C11–C13; maximum deviation = 0.045 (2) Å at atom C12].

In the crystal (Fig. 2), molecules are linked by a pair of N1—H1N1···O1 hydrogen bonds into an inversion dimer with an *R*_2_^2^(8) ring motif. The dimers are further connected by C10—H10A···N3 hydrogen bonds into an infinite chain along [101]. The crystal packing also features C—H···π interaction (Table 1), involving *Cg*1 which is the centroid of C5–C10 ring.

### Experimental

Equimolar amounts (0.01 mol) of tetralone and 2-cyanoacetohydrazide in dioxane (30 ml) were heated under reflux for 6 h. The mixture was then cooled at room temperature for overnight. The precipitated solid was filtered off, washed with ethanol, dried and crystallized from methanol to afford the title compound as colourless blocks with 73% abundance, *m.p.*: 183–185 °C.

### Refinement

The atom H1N1 was located in a difference fourier map and refined freely [N1—H1N1 = 0.90 (2) Å]. The remaining H atoms were positioned geometrically [C—H = 0.93 and 0.97 Å] and refined using a riding model with *U*_iso_(H) = 1.2 *U*_eq_(C).

### Crystal data

**Table:**

C_13_H_13_N_3_O	*Z* = 2
*M**_r_* = 227.26	*F*(000) = 240
Triclinic, *P*1	*D*_x_ = 1.297 Mg m^−^^3^
Hall symbol: -P 1	Cu *K*α radiation, λ = 1.54178 Å
*a* = 7.6414 (1) Å	Cell parameters from 2725 reflections
*b* = 7.6748 (1) Å	θ = 5.8–70.2°
*c* = 10.5644 (2) Å	µ = 0.69 mm^−^^1^
α = 109.589 (1)°	*T* = 296 K
β = 91.405 (1)°	Block, colourless
γ = 93.260 (1)°	0.59 × 0.51 × 0.40 mm
*V* = 582.13 (2) Å^3^	

### Data collection

**Table:**

Bruker SMART APEXII CCD diffractometer	1898 independent reflections
Radiation source: fine-focus sealed tube	1762 reflections with *I* > 2σ(*I*)
Graphite monochromator	*R*_int_ = 0.017
φ and ω scans	θ_max_ = 65.0°, θ_min_ = 5.8°
Absorption correction: multi-scan (*SADABS*; Bruker, 2009)	*h* = −8→8
*T*_min_ = 0.687, *T*_max_ = 0.771	*k* = −7→9
5749 measured reflections	*l* = −12→12

### Refinement

**Table:**

Refinement on *F*^2^	Secondary atom site location: difference Fourier map
Least-squares matrix: full	Hydrogen site location: inferred from neighbouring sites
*R*[*F*^2^ > 2σ(*F*^2^)] = 0.043	H atoms treated by a mixture of independent and constrained refinement
*wR*(*F*^2^) = 0.120	*w* = 1/[σ^2^(*F*_o_^2^) + (0.0686*P*)^2^ + 0.0904*P*] where *P* = (*F*_o_^2^ + 2*F*_c_^2^)/3
*S* = 1.05	(Δ/σ)_max_ < 0.001
1898 reflections	Δρ_max_ = 0.18 e Å^−^^3^
159 parameters	Δρ_min_ = −0.17 e Å^−^^3^
0 restraints	Extinction correction: *SHELXTL* (Sheldrick, 2008), Fc^*^=kFc[1+0.001xFc^2^λ^3^/sin(2θ)]^-1/4^
Primary atom site location: structure-invariant direct methods	Extinction coefficient: 0.030 (3)

### Special details

**Table:**

Geometry. All e.s.d.'s (except the e.s.d. in the dihedral angle between two l.s. planes) are estimated using the full covariance matrix. The cell e.s.d.'s are taken into account individually in the estimation of e.s.d.'s in distances, angles and torsion angles; correlations between e.s.d.'s in cell parameters are only used when they are defined by crystal symmetry. An approximate (isotropic) treatment of cell e.s.d.'s is used for estimating e.s.d.'s involving l.s. planes.
Refinement. Refinement of *F*^2^ against ALL reflections. The weighted *R*-factor *wR* and goodness of fit *S* are based on *F*^2^, conventional *R*-factors *R* are based on *F*, with *F* set to zero for negative *F*^2^. The threshold expression of *F*^2^ > σ(*F*^2^) is used only for calculating *R*-factors(gt) *etc*. and is not relevant to the choice of reflections for refinement. *R*-factors based on *F*^2^ are statistically about twice as large as those based on *F*, and *R*- factors based on ALL data will be even larger.

### Fractional atomic coordinates and isotropic or equivalent isotropic displacement parameters (Å^2^)

**Table:**

	*x*	*y*	*z*	*U*_iso_*/*U*_eq_	
O1	0.19129 (16)	0.4167 (2)	0.53245 (10)	0.0798 (4)	
N1	0.07938 (15)	0.36666 (18)	0.32370 (11)	0.0530 (3)	
N2	0.10531 (14)	0.29801 (15)	0.18782 (10)	0.0456 (3)	
N3	0.5764 (3)	0.2338 (4)	0.5360 (2)	0.1264 (9)	
C1	−0.02329 (16)	0.29510 (17)	0.10641 (13)	0.0419 (3)	
C2	−0.20191 (18)	0.3606 (2)	0.14809 (15)	0.0531 (4)	
H2A	−0.1954	0.4950	0.1788	0.064*	
H2B	−0.2357	0.3218	0.2230	0.064*	
C3	−0.34209 (18)	0.2862 (2)	0.03515 (16)	0.0590 (4)	
H3A	−0.3665	0.1544	0.0168	0.071*	
H3B	−0.4494	0.3470	0.0632	0.071*	
C4	−0.28501 (19)	0.3183 (2)	−0.09133 (16)	0.0583 (4)	
H4A	−0.2742	0.4504	−0.0759	0.070*	
H4B	−0.3738	0.2621	−0.1629	0.070*	
C5	−0.11171 (18)	0.23730 (19)	−0.13436 (14)	0.0491 (4)	
C6	0.01244 (16)	0.22607 (16)	−0.03809 (12)	0.0416 (3)	
C7	0.17256 (17)	0.15242 (19)	−0.08101 (14)	0.0478 (3)	
H7A	0.2555	0.1441	−0.0177	0.057*	
C8	0.2098 (2)	0.0923 (2)	−0.21424 (15)	0.0575 (4)	
H8A	0.3170	0.0438	−0.2407	0.069*	
C9	0.0876 (2)	0.1038 (2)	−0.30948 (15)	0.0636 (4)	
H9A	0.1122	0.0629	−0.4001	0.076*	
C10	−0.0706 (2)	0.1761 (2)	−0.26907 (15)	0.0613 (4)	
H10A	−0.1520	0.1842	−0.3334	0.074*	
C11	0.20438 (19)	0.3556 (2)	0.41121 (14)	0.0547 (4)	
C12	0.3655 (2)	0.2592 (2)	0.35112 (15)	0.0619 (4)	
H12A	0.3304	0.1361	0.2892	0.074*	
H12B	0.4251	0.3284	0.3011	0.074*	
C13	0.4837 (2)	0.2454 (3)	0.45556 (17)	0.0712 (5)	
H1N1	−0.010 (3)	0.434 (3)	0.3642 (19)	0.078 (5)*	

### Atomic displacement parameters (Å^2^)

**Table:**

	*U*^11^	*U*^22^	*U*^33^	*U*^12^	*U*^13^	*U*^23^
O1	0.0795 (8)	0.1259 (11)	0.0388 (6)	0.0515 (7)	0.0104 (5)	0.0263 (6)
N1	0.0473 (7)	0.0739 (8)	0.0390 (6)	0.0203 (6)	0.0055 (5)	0.0178 (5)
N2	0.0432 (6)	0.0558 (7)	0.0378 (6)	0.0092 (5)	0.0022 (4)	0.0148 (5)
N3	0.0968 (14)	0.189 (2)	0.0923 (14)	0.0546 (15)	−0.0274 (11)	0.0414 (14)
C1	0.0377 (7)	0.0438 (7)	0.0456 (7)	0.0036 (5)	0.0000 (5)	0.0167 (5)
C2	0.0420 (7)	0.0643 (9)	0.0538 (8)	0.0104 (6)	0.0054 (6)	0.0195 (7)
C3	0.0374 (7)	0.0665 (9)	0.0744 (10)	0.0053 (6)	−0.0015 (6)	0.0255 (8)
C4	0.0441 (8)	0.0667 (9)	0.0653 (9)	0.0060 (6)	−0.0122 (6)	0.0245 (7)
C5	0.0452 (7)	0.0495 (7)	0.0512 (8)	0.0002 (6)	−0.0089 (6)	0.0164 (6)
C6	0.0395 (7)	0.0401 (6)	0.0429 (7)	0.0007 (5)	−0.0037 (5)	0.0117 (5)
C7	0.0438 (7)	0.0517 (7)	0.0450 (7)	0.0076 (6)	−0.0041 (5)	0.0122 (6)
C8	0.0544 (8)	0.0603 (9)	0.0503 (8)	0.0106 (7)	0.0047 (6)	0.0078 (7)
C9	0.0711 (10)	0.0723 (10)	0.0396 (7)	0.0049 (8)	−0.0002 (7)	0.0089 (7)
C10	0.0632 (9)	0.0711 (10)	0.0466 (8)	0.0019 (7)	−0.0144 (7)	0.0174 (7)
C11	0.0542 (8)	0.0729 (9)	0.0398 (7)	0.0209 (7)	0.0061 (6)	0.0197 (6)
C12	0.0528 (8)	0.0840 (11)	0.0442 (8)	0.0228 (7)	0.0006 (6)	0.0127 (7)
C13	0.0574 (9)	0.0923 (12)	0.0596 (9)	0.0241 (8)	−0.0055 (8)	0.0175 (9)

### Geometric parameters (Å, º)

**Table:**

O1—C11	1.2163 (17)	C4—H4B	0.9700
N1—C11	1.3389 (18)	C5—C10	1.391 (2)
N1—N2	1.3770 (15)	C5—C6	1.4009 (18)
N1—H1N1	0.90 (2)	C6—C7	1.3986 (19)
N2—C1	1.2841 (16)	C7—C8	1.369 (2)
N3—C13	1.122 (2)	C7—H7A	0.9300
C1—C6	1.4766 (18)	C8—C9	1.383 (2)
C1—C2	1.5061 (18)	C8—H8A	0.9300
C2—C3	1.519 (2)	C9—C10	1.376 (2)
C2—H2A	0.9700	C9—H9A	0.9300
C2—H2B	0.9700	C10—H10A	0.9300
C3—C4	1.508 (2)	C11—C12	1.5140 (19)
C3—H3A	0.9700	C12—C13	1.444 (2)
C3—H3B	0.9700	C12—H12A	0.9700
C4—C5	1.510 (2)	C12—H12B	0.9700
C4—H4A	0.9700		
			
C11—N1—N2	119.64 (11)	C6—C5—C4	120.20 (13)
C11—N1—H1N1	112.9 (12)	C7—C6—C5	118.82 (12)
N2—N1—H1N1	127.0 (12)	C7—C6—C1	120.57 (11)
C1—N2—N1	117.97 (11)	C5—C6—C1	120.59 (11)
N2—C1—C6	116.02 (11)	C8—C7—C6	121.36 (12)
N2—C1—C2	124.94 (12)	C8—C7—H7A	119.3
C6—C1—C2	119.01 (11)	C6—C7—H7A	119.3
C1—C2—C3	112.97 (12)	C7—C8—C9	119.94 (13)
C1—C2—H2A	109.0	C7—C8—H8A	120.0
C3—C2—H2A	109.0	C9—C8—H8A	120.0
C1—C2—H2B	109.0	C10—C9—C8	119.50 (14)
C3—C2—H2B	109.0	C10—C9—H9A	120.3
H2A—C2—H2B	107.8	C8—C9—H9A	120.3
C4—C3—C2	111.41 (12)	C9—C10—C5	121.62 (13)
C4—C3—H3A	109.3	C9—C10—H10A	119.2
C2—C3—H3A	109.3	C5—C10—H10A	119.2
C4—C3—H3B	109.3	O1—C11—N1	122.97 (13)
C2—C3—H3B	109.3	O1—C11—C12	120.79 (13)
H3A—C3—H3B	108.0	N1—C11—C12	116.23 (12)
C3—C4—C5	111.56 (12)	C13—C12—C11	110.54 (13)
C3—C4—H4A	109.3	C13—C12—H12A	109.5
C5—C4—H4A	109.3	C11—C12—H12A	109.5
C3—C4—H4B	109.3	C13—C12—H12B	109.5
C5—C4—H4B	109.3	C11—C12—H12B	109.5
H4A—C4—H4B	108.0	H12A—C12—H12B	108.1
C10—C5—C6	118.76 (13)	N3—C13—C12	179.4 (2)
C10—C5—C4	121.04 (12)		
			
C11—N1—N2—C1	173.62 (13)	C2—C1—C6—C7	−175.60 (12)
N1—N2—C1—C6	178.06 (11)	N2—C1—C6—C5	−172.51 (11)
N1—N2—C1—C2	−0.3 (2)	C2—C1—C6—C5	5.95 (18)
N2—C1—C2—C3	−161.63 (13)	C5—C6—C7—C8	0.2 (2)
C6—C1—C2—C3	20.06 (18)	C1—C6—C7—C8	−178.26 (12)
C1—C2—C3—C4	−50.51 (17)	C6—C7—C8—C9	0.0 (2)
C2—C3—C4—C5	55.23 (17)	C7—C8—C9—C10	0.1 (2)
C3—C4—C5—C10	151.26 (14)	C8—C9—C10—C5	−0.4 (3)
C3—C4—C5—C6	−30.03 (18)	C6—C5—C10—C9	0.6 (2)
C10—C5—C6—C7	−0.5 (2)	C4—C5—C10—C9	179.32 (15)
C4—C5—C6—C7	−179.24 (12)	N2—N1—C11—O1	178.53 (15)
C10—C5—C6—C1	177.98 (12)	N2—N1—C11—C12	−2.2 (2)
C4—C5—C6—C1	−0.76 (19)	O1—C11—C12—C13	3.3 (2)
N2—C1—C6—C7	5.94 (18)	N1—C11—C12—C13	−175.97 (15)

### Hydrogen-bond geometry (Å, º)

Cg1 is the centroid of the C5–C10 ring.

**Table:**

*D*—H···*A*	*D*—H	H···*A*	*D*···*A*	*D*—H···*A*
N1—H1*N*1···O1^i^	0.91 (2)	1.96 (2)	2.8640 (17)	174.7 (19)
C10—H10*A*···N3^ii^	0.93	2.58	3.491 (3)	167
C2—H2*A*···*Cg*1^iii^	0.97	2.80	3.6775 (17)	152

Symmetry codes: (i) −*x*, −*y*+1, −*z*+1; (ii) *x*−1, *y*, *z*−1; (iii) −*x*, −*y*+1, −*z*.
